# Bacteria of healthy periodontal tissues as candidates of probiotics: a systematic review

**DOI:** 10.1186/s40001-024-01908-2

**Published:** 2024-06-14

**Authors:** María del Pilar Angarita-Díaz, Cristian Fong, Daniela Medina

**Affiliations:** 1 GIOMET Group, Faculty of Dentistry, Universidad Cooperativa de Colombia, Campus Villavicencio, Carrera 35 # 36 99, Villavicencio, Colombia; 2 Ciencia y Pedagogía Group, School of Medicine, Universidad Cooperativa de Colombia, Campus Santa Marta, Santa Marta, Colombia; 3 School of Dentistry, Universidad Cooperativa de Colombia, Campus Villavicencio, Villavicencio, Colombia

**Keywords:** Bacteria, Microbiota, Oral health, Periodontium, Probiotics, *Streptococcus*, Symbiosis

## Abstract

**Objectives:**

The use of probiotics could promote the balance of the subgingival microbiota to contribute to periodontal health. This study aimed to identify the potential of bacteria commonly associated with healthy periodontal tissues as probiotic candidates.

**Material and methods:**

A systematic review was conducted according to the Preferred Reporting Items for Systematic Reviews and Meta-Analyses guidelines using the PubMed, Scopus, Science Direct, ProQuest, and Ovid databases as well as the combination of Medical Subject Headings (MeSH) and non-MeSH terms. Based on the selection criteria, original studies published in English and identifying the microorganisms present in the periodontium of healthy individuals and patients with periodontitis using the high-throughput 16S ribosomal gene sequencing technique were included.

**Results:**

Out of 659 articles, 12 met the criteria for this review. These articles were published from 2012 to 2020 and mainly originated from the United States, China, and Spain. Most of these studies reported adequate criteria for selecting participants, using standardized clinical criteria, and compliance with quality based on the tools used. In periodontal healthy tissue were identified species like *Actinomyces viscosus*, *Actinomyces naeslundii, Haemophilus parainfluenzae, Rothia dentocariosa, Streptococcus sanguinis, Streptococcus mitis, Streptococcus oralis, Streptococcus gordonii, Streptococcus intermedius, and Prevotella nigrescens* which have recognized strains with a capacity to inhibit periodontopathogens*.*

**Conclusions:**

*S. sanguinis*, *S. oralis*, *S. mitis*, and *S. gordonii* are among the bacterial species proposed as potential probiotics because some strains can inhibit periodontopathogens and have been reported as safe for humans.

**Supplementary Information:**

The online version contains supplementary material available at 10.1186/s40001-024-01908-2.

## Introduction

Periodontitis is a chronic and inflammatory disease of bacterial etiology that results in loss of periodontal attachment [1]. In periodontitis, the gums detach from the teeth, and the supporting tissues are destroyed in the most advanced and severe stages, thereby leading to bone and tooth loss [2]. Moreover, this disease has been associated with several other important conditions, such as diabetes, cardiovascular diseases, and Alzheimer’s disease [3]. Periodontitis affects approximately 50% of the adult population worldwide [4] with varying degrees of severity and approximately 11% of this population has severe periodontitis [5]. In Colombia, 61.8% of the population has periodontitis, and 10.6% of cases are classified as severe periodontitis [6].

Microbiological and molecular studies were used to distinguish the microbiota associated with healthy periodontium from those associated with diseased periodontium [7]. For example, gram-positive and some gram-negative bacteria have been associated with a healthy state. *Actinomyces* and *Streptococcus* species are the first colonizers of pristine tooth surfaces and co-aggregate to form early dental biofilm. Notably, bacteria identified in healthy periodontal tissue samples include *Peptostreptococcus*, *Gemella*, *Veillonella, Capnocytophaga*, *Neisseria*, *Rothia*, and *Corynebacterium* [7–9]. Bacteria such as *Porphyromonas gingivalis*, *Tannerella forsythia*, and *Treponema denticola* have been identified in patients with periodontal disease. In addition, these patients had bacteria characterized by the presence of virulence factors associated with alteration in the tissue immune response (e.g., activation of destructive proinflammatory patterns or evasion of the immune response) and destruction of periodontal tissues (e.g., by lytic enzymes), promoting the onset and development of severe diseases [9, 10].

Other microbiological features of periodontitis are the presence of a greater diversity of bacteria and significant differences between the functional profiles of these microorganisms and those of healthy periodontium. In addition, a decrease, but not disappearance, in the proportion of some bacterial genera and species associated with healthy periodontium has been observed [9, 11]. These properties reflect the approach to disease onset: instead of a few pathogens, a dysbiotic polymicrobial community is considered to generate harmful inflammatory responses that perpetuate the imbalance or dysbiosis of the microbiota and its environment [11].

In addition to the accumulation of a diverse bacterial population in the sulcus of the gingiva, other risk factors are known to be involved in the occurrence of dysbiosis in systemically healthy individuals. These include unhealthy diet, smoking, and other habits that alter the environment of the tissues, i.e., the availability of oxygen and nutrients [12]. This promotes the emergence of a range of microorganisms, mainly bacteria, which act synergistically to enhance their metabolic activities, causing changes in the composition of the microbiota and thereby damaging periodontal tissues [7, 11, 13, 14].

Among the novel treatments being investigated for controlling periodontitis and maintaining a healthy periodontium, probiotics have been proposed as adjuvants to traditional treatments. Probiotics include live beneficial microorganisms that benefit the host when administered in sufficient quantity because they have properties that inhibit pathogenic microorganisms through the production of antimicrobial components or competition for host nutrients or tissue-binding sites. Furthermore, they may have immunomodulation properties, further strengthening the immune response [15].

The bacterial genera *Lactobacillus* and *Bifidobacterium* are most widely studied as probiotics to prevent periodontitis or as adjuvants during its treatment. Notably, these bacteria can inhibit periodontal pathogens and are safe for human consumption. However, even after examining the same probiotic strains, not all studies have discovered beneficial or consistent outcomes in clinical indices and/or in controlling the pathogenic microbiota [16–18]. This can be explained by various factors, such as the different administration routes, doses, and quantities; combinations with other probiotics; administration times; and/or sample sizes of clinical studies [16–18]. In addition, these bacteria are not normally abundant in periodontal tissues [8, 11], and some bacteria are of intestinal origin, which may affect their ability to colonize the oral cavity [19]. This further limits their effectiveness as probiotics; thus, further studies are needed to identify or investigate probiotics suitable for this tissue and periodontal health.

Bacteria that are more frequently in the microbiota of the healthy periodontium and have probiotic characteristics could be analyzed as possible probiotic candidates [20, 21]. Therefore, this review aimed to identify bacteria with probiotic characteristics in the microbiota of the healthy periodontium for treating periodontitis.

## Material and methods

### Type of study and search strategy

We conducted a systematic review of the literature as per the updated quality criteria of the Preferred Reporting Items for Systematic Reviews and Meta-Analyses statement. The PubMed, ScienceDirect, Scopus, and Ovid databases were used to search for articles from July 2012 to April 2022 using both Medical Subject Headings (MeSH) and non-MeSH terms (Table 1); in addition, the associated filters for year and type of study were used. Furthermore, we conducted a manual search of information.

**Table 1:** Keywords and combinations according to the database

Database	Search strategy
PubMed	((**Periodontitis** OR “**Chronic Periodontitis**” OR “Periodontal disease” OR “Gum disease”) NOT (children OR “Alzheimer’s disease” OR Pregnant OR smoker)) AND (**Microbiology** OR **Bacteria** OR Bacterium) AND (Microbial balance OR **Symbiosis** OR **Dysbiosis**) AND (Identification OR Recognition OR Detection OR **Prevalence**)
PubMed	(Subgingival) AND (**microbiota** OR microbiome) AND (“periodontal health” OR **health**) AND (**periodontitis**)
Ovid	(**Periodontitis** OR “**Chronic Periodontitis**” OR “Periodontal disease” OR “Gum disease “) AND (**Microbiology** OR **Bacteria** OR Bacterium) AND (Microbial balance OR **Symbiosis** OR **Dysbiosis**) AND (Identification OR Recognition OR Detection OR **Prevalence**) NOT (children OR “Alzheimer’s disease” OR Pregnant OR smoker)
ScienceDirect	(**Periodontitis**) AND (human) AND (**bacteria**) AND (**symbiosis** OR microbial balance OR **dysbiosis**) AND (identification) NOT (“Alzheimer’s disease” OR **HIV**)
ScienceDirect	(**Periodontitis** OR “periodontal disease”) AND (subgingival) AND (microbiome OR **microbiota**) AND (profile)
Scopus	(**Periodontitis** OR “**Chronic Periodontitis**” OR “Periodontal disease” OR “Gum disease”) AND NOT (children OR “Alzheimer’s disease” OR Pregnant OR smoker)) AND (**Microbiology** OR **Bacteria** OR Bacterium) AND (Microbial balance OR **Symbiosis** OR **Dysbiosis**) AND (Identification OR Recognition OR Detection OR **Prevalence**)
Scopus	(**Periodontitis** OR “periodontal disease”) AND (subgingival) AND (microbiome OR **microbiota**) AND (profile)

MeSH terms are indicated using bold font

The PICO format, Population (healthy population), Intervention (metagenomic analysis), Comparison (patients with periodontitis), Outcome (group of potential probiotics for the treatment of periodontitis), was used to formulate our clinical question: Which bacteria with probiotic characteristics for the treatment of periodontitis predominate in the microbiota of the periodontium of healthy patients? Inclusion criterion was original studies published in English that identified microorganisms present in subgingival tissue samples of healthy individuals using the high-throughput sequencing technique of the 16S ribosomal gene. Studies that did not identify bacteria in samples from healthy adults and patients with periodontitis in the same analysis and articles that did not report the nationality and/or sex of the patients were excluded. In addition, we selected studies that excluded patients with systemic diseases, those treated with antibiotics or other types of drugs, or those who were receiving periodontal treatment for at least 3 months before collecting the subgingival tissue samples.

### Selection of the studies and collection of data

First, studies with unique titles were identified, their abstracts were read, and, eventually, those related to the purpose of the present study were selected. The selected studies were then thoroughly read to verify compliance with the selection criteria, leaving the final articles for review. This process was carried out by two researchers (MPAD and DM).

Finally, the studies were synthesized using the defined variables, including authors, origin, and type of the study; clinical parameters of the patients; selection criteria; analyzed samples; amplified sequence; and periodontal tissue bacteria identified in healthy individuals.

### Level of evidence and risk of bias

The quality of the articles included in this review was assessed by two researchers (MPAD and CF) using the Joanna Briggs Institute (JBI) scale for case–control and cross-sectional studies. This scale enabled the evaluation of reliability, relevance, and results of the selected articles.

## Results

### Selection of studies and characteristics of the studies

**Fig. 1:**
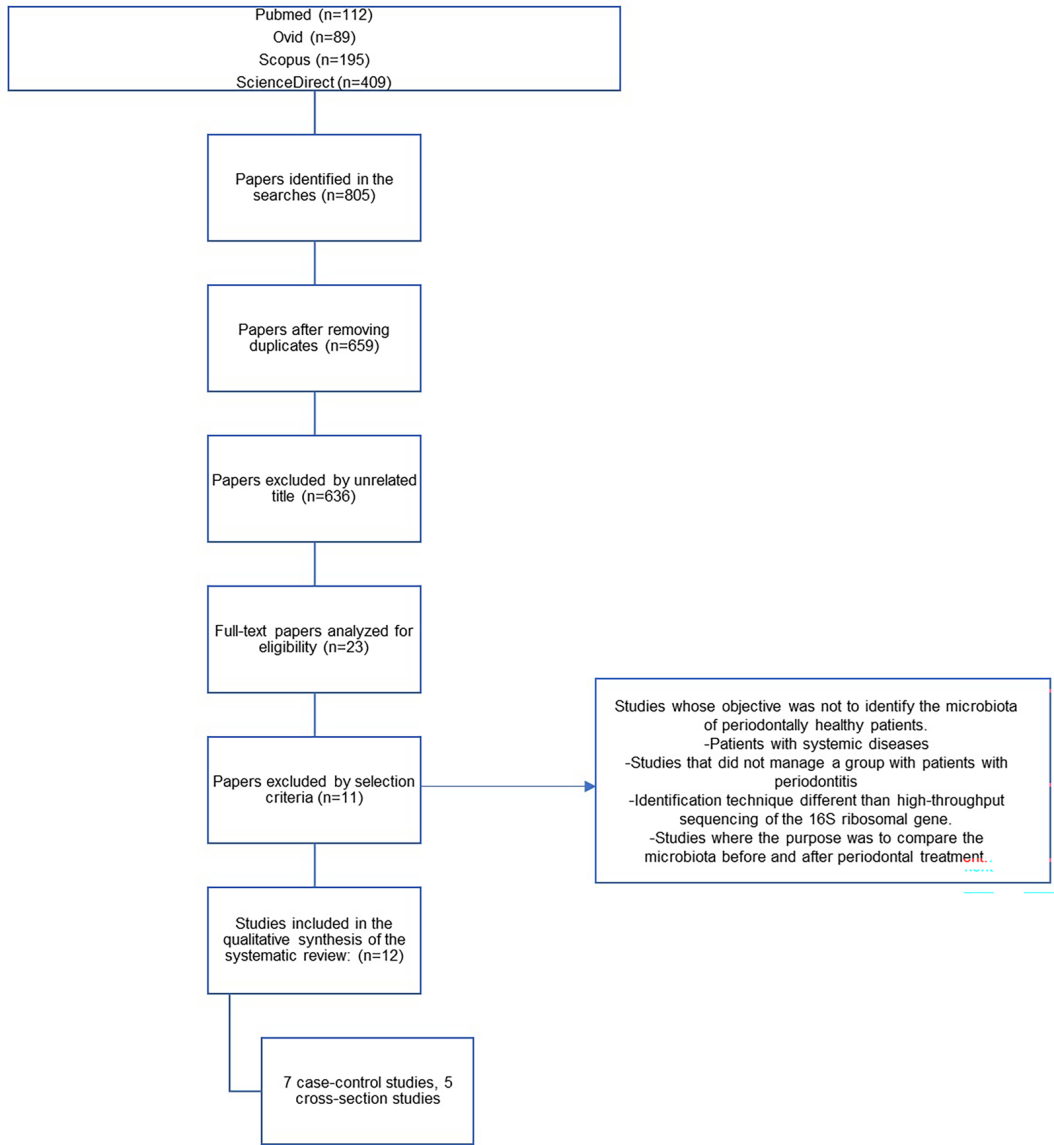
Information flowchart of the different stages of a systematic review

Overall, 659 unique articles were found based on the information search; of these, 23 were selected for full reading, and 12 that met the selection criteria were eventually selected for review (Fig. 1). Most investigations were conducted in the United States (*n* = 4), followed by China (*n* = 2) and Spain (*n* = 2). The remaining four studies were conducted in Chile, Germany, Japan, and the Czech Republic (Table 2). The most common study types were case–control (58.3%, *n* = 7) and cross-sectional (41.6%, *n* = 5) studies (Fig. 1). Neither of the selected studies was a randomized clinical trial (Table 2).

**Table 2:** Characteristics of the included studies

Author/year/country	Type of study	Patients	Clinical parameters	Selection criteria	Sample analyzed	Amplified region	Bacteria identified and abundant in periodontal health
Griffen, et al./2012/United States [22]	Case–control	-29 healthy individuals (54.1 ± 12.4 years) -29 patients with chronic periodontitis (51.3 ± 12.6 years)	- Healthy individuals: PD: < 4 mm Patients with chronic periodontitis: CAL: ≥ 4 mm and PD: ≥ 5 mm in at least three nonadjacent interproximal sites in at least two quadrants	- Minimum age of 35 years -Minimum of 20 teeth -No antibiotic or periodontal therapy for the last 3 months -No steroid or immunosuppressant medication and no history of diabetes or HIV	Healthy individuals and patients with periodontitis: Subgingival tissue samples collected using paper points inserted at three sites for 10 s	V1–V2 and V4	-Higher proportion of proteobacteria -Abundant presence of the genus *Streptococcus* (*S. sanguinis, S. mitis*, *S. intermedius, S. oralis* taxon B66), followed by *Acinetobacter* (*A. junii, A.* sp. RUH1139), *Moraxella* (*M. osloensis*), *Haemophillus* ( *H*. *parahaemolyticus*, *H.* P3D1620, H. P3D1 620), *Granulicatella* (*G*. *adiacens*), *Actinomyces* (*A. viscosus naeslundii*, *A. massiliensis*, *A.* oral taxon 171), *Aggregatibacter*, *Rothia* (*R. dentocariosa*, *R. aeria*), *Arthrobacter* (*A. woluwensis*), *Gemella* (*G. morbillorum*), *Lautropia* (*L.* ap009, L. *mirabilis*), unclassified *Comamonadaceae* (C. nbu379c11c1, C. VE3A04, C. 9863833), *Brachybacterium* (*B. rhamnosus*)
Kumar et al./2012/United states [23]	Case–control	-10 healthy individuals (35.5 ± 7.5) -10 patients with periodontitis (42.5 ± 8.2)	Healthy individuals: PD: ≤ 3 mm, CAL: ≤ 1 mm Patients with chronic periodontitis: CAL: ≥ 4 mm in more than 30% of the sites	-Patients with at least 20 teeth -Patients who do not present with diabetes, pregnancy, or HIV infection -Patients who have not been treated with antibiotics; medications, such as immunosuppressants, bisphosphonates, or steroids; or periodontal treatment during the last 3 months	-Subgingival sample collected with paper points within the sulcus for 10 s -Healthy individuals: Sample of the mesial sulcus of 15 randomly selected teeth -Patients with periodontitis: Sample of four nonadjacent proximal sites that presented at least 6 mm of CAL and 5 mm of PD	V1–V3 and V7–V9	-Increased presence of gram-positive aerobic bacteria -Specific presence of the following: *Kingella, Aggregatibacter*, and *Centipeda*
Abusleme et al./2013/Chile[24]	Cross-sectional	-10 Healthy individuals (34.1 ± 5.5 years) -22 patients with chronic periodontitis (42.5 ± 3.3 years)	- Healthy individuals: PD and CAL: ≤ 3 mm in more than 90% of sites, and less than 10% of sites with BOP **-** Patients with chronic periodontitis: PD: ≥ 5 mm; CAL: ≥ 4 mm; BOP in at least 20% of sites showing bone loss on radiograph	-Systemically healthy individuals -Nonsmoking patients -Patients without pregnancy -Patients without periodontal treatment, antibiotic treatment, anticoagulants, or nonsteroidal anti-inflammatory drugs in the last 6 months -Patients with at least 14 natural teeth	- Healthy individuals: Subgingival tissue sample collected using curette from two sites without BOP - Tissue samples of patients with periodontitis: Subgingival biofilm curetted from two nonadjacent sites with a similar amount of periodontal destruction but different BOP	V1–V2	- Significantly most abundant genera: *Actinomyces*, *Rothia* -Other abundant genera: *Burkholderia, Corynebacterium* -Predominance of OTUs associated with *Actinomyces* spp., *Streptococcus* spp. (related to *S. sanguinis), Proteobacteria*, and *Porphyromonas* spp. (related to *P. catoniae*)
Li et al./2014/Chin [25]	Case–control	-12 Healthy individuals -12 patients with moderate periodontitis -13 patients with severe periodontitis	-Healthy individuals: Without PAL, CAL, or alveolar destruction; < 15% of sites with BOP -Patients with moderate periodontitis: PD: 4–6 mm; CAL: 3–5 mm; destruction of the alveolar bone: 1/3–1/2 of the root length -Patients with severe periodontitis: PD: ≥ 6 mm; CAL: > 5 mm; destruction of the alveolar bone: > 1/2 of the length of the root	- Patients between 20 and 70 years -Systemically healthy individuals -No periodontal treatment or antibiotic treatment in the last 6 months	- Healthy individuals and patients with periodontal disease: Subgingival tissue sample collected using sterile paper points left for 10 s in selected periodontal pockets	V4	- Higher proportion of proteobacteria - Most significantly abundant genera: *Neisseria, Corynebacterium.* Other genera:* Leptotrichia*
Kirst et al./ 2015/ United States [26]	Case–control	-25 periodontally healthy individuals -25 patients with chronic periodontitis	- Healthy individuals: CAL: ≤ 3 mm -Patients with chronic periodontitis: CAL: ≥ 5 mm	-No history of systemic diseases that may interfere with clinical features, incidence, or disease progression -No periodontal treatment in the last 6 months -No treatment with any medication that affects the periodontal status within the last 3 months (antibiotics, anti-inflammatory drugs, contraceptives)	-Healthy individuals and patients with periodontitis: Subgingival tissue sample collected using paper points placed deep in the sulcus and moving them laterally along the tooth surface and epithelial lining of the sulcus from two different patient sites	V1–V3	-The families *Streptococcaceae, Fusobacteriaceae, Micrococcaceae*, and *Actinomycetaceae* were predominant - Predominance of OTUs: *Actinomyces* oral taxon 170, *S. mitis, S. sanguis, Gemella haemolysans,* and* G. adiacens*
Camelo-Castillo, et al./ 2015/Spain [27]	Case–control	-22 healthy nonsmokers (44.95 ± 13.04 years) -28 nonsmokers with periodontitis (54.14 ± 11.9 years) -32 smokers with periodontitis (49.19 ± 7.46)	Healthy individuals: PD: < 4 mm, without radiographic evidence of alveolar bone loss and BOP < 20% -Patients with chronic periodontitis: moderate: ≥ 2 interproximal sites with CAL ≥ 4 mm or ≥ 1 site with PD ≥ 5 mm -Patients with severe periodontitis: ≥ 2 interproximal sites with CAL ≥ 6 mm and ≥ 1 site with PD ≥ 5 mm	-30–65 years -Good general health -No pregnancy and lactation status -No antibiotic treatment in the previous 6 months -No treatment with anti-inflammatory drugs in the previous 4 months -No use of oral antiseptics -No appliances and implantology -No periodontal treatment -At least 18 natural teeth	- Healthy individuals: Subgingival tissue sample collected using paper points in each gingival sulcus of eight teeth in quadrants 1 and 3 (incisor, canine, premolar, and molar) - Patients with periodontitis: Subgingival tissue sample collected using sterile paper points left for 10 s in the gingival sulcus of the sites with greater depth to probing in eight nonadjacent proximal sites	V1–V3	- Most abundant species: *Fusobacterium nucleatum* - Significantly abundant genera: *Capnocytophaga, Corynebacterium, Gemella, Haemophilus, Leptotrichia, Streptococcus*, and* Veillonella* - Significantly high prevalence of positive patients for genera: *Abiotrophia, Aggregatibacter, Cardiobacterium, Clostridium, Corynebacterium, Eikenella, eubacterium, Granulicatella, haemophilus, Kingella, Neisseria, Ottowia, Propionibacterium, Propionivibrio, Rothia, Schlegelella, Tessaracoccus*
Chen et al./2018/United States [28]	Cross-sectional	-21 healthy individuals (31.9 [19–79] years) -48 adults with chronic periodontitis (52.0 [25–79] years)	- Healthy individuals: Without CAL or BOP. PD ≤ 3 mm in all teeth, except the third molar - Patients with chronic periodontitis: PD and CAL ≥ 3 mm in at least 30% of the teeth	- No antibiotic treatment or periodontal treatment in the 6 months before sample collection -No medical conditions that may affect the immune response -No antibiotic medication before treatment -No pregnancy or lactation status -No medication that may affect the characteristics of subgingival bacteria	- Healthy individuals and patients with periodontitis: Subgingival tissue sample collected using a paper point for 10 s from two contralateral maxillary posterior teeth	V4	- Significant abundance of: *Actinobacteria* *Actinomyces, Veillonella, Capnocytophaga, Leptotrichia, Exiguobacterium, Paludibacter, Corynebacterium, Prevotella, Opitutus*
Schulz et al./2019/Germany [29]	Case–control	-13 patients with healthy periodontal tissue: 66.7% former smokers and 57.1% smokers; mean age. 40.8 ± 7.3 years -13 patients with aggressive periodontitis: 20.0% former smokers and 27.3% smokers; mean age, 45.9 ± 9.9 years	-Healthy patient: > 30 years old, PD ≤ 3.5 mm and without gingival resection -Patient with aggressive periodontitis: age at onset of the disease < 35 years, CAL ≥ 4 mm in at least 30% of the teeth, where at least three of the affected teeth are not first molars or incisors	-No pregnancy status -No drug-induced gingival hyperplasia -No antibiotic treatment in the last 6 months -No periodontal therapy in the last 3 months -No HIV, acute infection of the oral cavity, oral pemphigus or pemphigoid, type I or type II diabetes mellitus, coronary heart disease, rheumatic diseases, lupus erythematous, Behcet’s disease, or Crohn’s disease	- Healthy and periodontitis patients: Subgingival tissue sample from the deepest pocket of each quadrant obtained using a sterile paper tip for 20 s	V3–V4	- On an average, Proteobacteria and Firmicutes were the most common phyla - Bacteria associated with healthy periodontal tissue: *Neisseria lactamica, L. mirabilis, Corynebacterium matruchotti, Rothia dentocariosa, Acinetobacter gerneri, Veillonella atypica, Actinomyces meyeri, Streptococcus intermedius, Streptococcus tigurinus, Mannheimia caviae, Campylobacter gracilis, Streptococcus sanguinis, Acinetobacter antiviralis, Acinetobacter baumannii, Haemophilus parainfluenzae, Acinetobacter rhizosphaerae, Acinetobacter calcoaceticus*
López-Martínez et al./ 2020/Spain [30]	Cross-sectional clinical study	-10 Periodontally healthy individuals (47 [36–57] years) -12 patients with chronic periodontitis (52 [34–69] years)	-Healthy patient: minimum 20 teeth without BOP; CAL < 4 mm -Patients with periodontitis: CAL ≥ 4 mm and PD > 5 mm in at least three adjacent interproximal sites; more than 20% of sites with BOP	- Nonsmoking patients -No systemic diseases -No antibiotic therapy for the last 6 months -No dental cleaning during the last 6 months	- Healthy and periodontitis patients: Subgingival tissue sample of periodontal pocket obtained using paper points inserted for 30 s	V1–V3	*-*High proportion of Phylum Actinobacteria Class Gammaproteobacteria - Most frequent genera; *Actinomyces* and *Rothia* Most frequent species*: R. dentocariosa*
Ikeda et al./2020/Japan [31]	Case–control	-10 Healthy individuals (60.3 ± 9.6) -10 patients with chronic periodontitis (65.2 ± 13.1)	- Healthy individuals: PD ≤ 3 mm at all sites -Patients with periodontitis: at least four sites with PD ≥ 6 mm in each quadrant	-Over 40 years old -With more than 20 natural teeth -Without the use of antibiotics and other antimicrobial agents, immunosuppressants, steroids, anti-inflammatory drugs, or drugs of biological origin in the 3 months before the study -No professional dental cleaning -No history of periodontal treatment	- Healthy and periodontitis patients: Subgingival sample of the periodontal pocket from the mesial sites of the maxillary first molar collected using paper points inserted for 30 s	V1–V2	Significantly abundant phyla: Actinobacteria and Proteobacteria Most abundant genera: *Rothia*, *Neisseria*, and *Leptotrichia* Most significantly abundant and prevalent species: *Neisseria subflava, Capnocytophaga gingivalis, Porphyromonas catoniae, Rothia aeria*, *Streptococcus infantis, Streptococcus oralis, Terrahaemophilus aromaticivorans,* and *Veillonella sp.* Most nonsignificantly abundant and prevalent species: *Lautropia mirabilis, Leptotrichia sp., Rothia dentocariosa, Eikenella corrodens, Neisseriaelongata, Neisseria sp., Propionibacterium propionicum,* and *Streptococcus gordonii*
Lenartova et al./2021/ Czech Republic [8]	Cross-sectional study	-91 healthy individuals (23 [19–38] years) -17 periodontally healthy individuals older than 40 years (46 [40–53] years) -45 patients with advanced periodontitis (33 [20–47] years)	- Healthy individuals: PD and CAL < 2 mm, no BOP, no signs of inflammation - Patients with severe periodontitis: at least two periodontal pockets with PD > 5 mm, confirmation of the diagnosis of severe periodontitis	Healthy individuals and patients with periodontitis: no antibiotic treatment or periodontal treatment 3 months before starting the study Patients with periodontitis: no history of treatment for periodontitis	- Subgingival tissue sample collected using sterile paper tips left for 10 s - Healthy individuals: buccal side of the gingival sulcus -Patients with periodontitis: deeper periodontal pocket	V4–V5	Most abundant and prevalent taxa: *Streptococcus mitis/S. oralis, CT43 Streptococcus gordonii/S. sanguinis, CT6 Veillonella dispar/V. parvula, CT25 Neisseria flava/N. mucosa, CT27 Neisseria subflava, HMT14 Neisseria oralis, HMT718 Haemophilus parainfluenzae, CT23 Haemophilus haemolyticus, CT24 H. sputorum, CT10 Prevotella histicola, CT37, Gemella morbillorum, CT48 Rothia dentocariosa, HMT22 Lautropia mirabilis, HMT37 Stenotrophomonas nitritireducens,* and* CT13 Aggregatibacter aphrophilus*
Lu et al./2021/China[32]	Cross-sectional study	Young adults aged between 18 and 21 years -15 patients with periodontitis (19.2 ± 0.9 years), 38 patients with gingivitis (stage 1: 19.0 ± 1.0 years, stage 2: 19.0 ± 1.5 years), and 15 periodontally healthy individuals (19.0 ± 0.0 years)	Healthy individuals: PD ≤ 3 mm, BOP < 10%, no CAL Gingivitis patients: intact periodontium, BOP ≥ 10%, no CAL Patients with stage I and II periodontitis diagnosis according to the new classification (2018), CAL ≤ 4 mm, and PD ≤ 5 mm	- More than 10 natural teeth -Nonsmokers -No systemic diseases -No oral appliances -No cavities or dentures on the selected teeth - No periodontal treatment during the 6 months before the study -Without treatment with antibiotics, anti-inflammatory drugs, or anticoagulants in the previous 3 months -No pregnancy or lactation	Sample tissues collected using curettes from the mesiobuccal and sublingual sites of the teeth 16, 26, 36, 46, 11, and 31	V3–V4	*Prevotella nigrescens,* Clostridiales F-3G-1876

*PD* probing depth; *CAL* clinical attachment level/loss; *BOP* Bleeding on probing; *OTUs* operational taxonomic units

Regarding patient selection, in addition to the exclusion criteria established in this study, some authors excluded pregnant and lactating patients, patients who had recently undergone prophylactic procedures, patients receiving oral antiseptics, smokers (only one study), patients with a certain number of natural teeth, etc. (Table 2).

Clinical criteria for the selection of healthy individuals were as follows: probing depth (PD) of < 2 to < 4 mm, clinical attachment level/loss (CAL) of 0 to < 4 mm, no bleeding on probing (BOP) or BOP in at least 10–20% of measured sites, no radiographic evidence of alveolar bone loss, and/or no evidence of inflammation. In most studies, samples were collected using paper points (66.7%, *n* = 8), whereas only two studies used curettes for sample collection (Table 2).

Regarding the most used amplified regions to identify bacteria, V1–V3 were predominantly used, followed by V4 (Table 2). Other regions used were V1–V2, V3, V3–V4, V4–V5, and V7–V9.

### Bacteria of periodontal healthy tissue

**Fig. 2:**
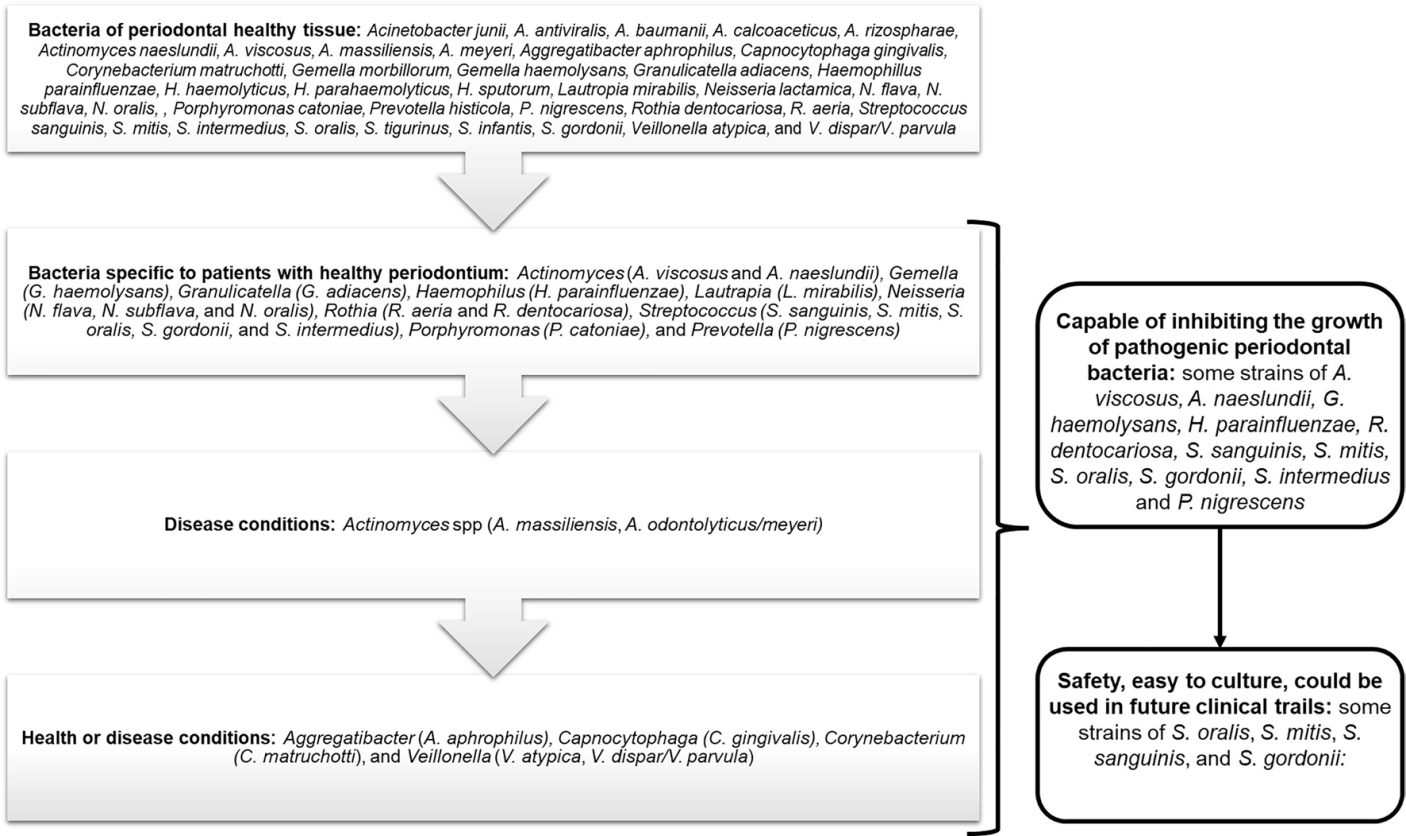
Summary of bacteria of healthy periodontal tissues as candidates of probiotics

Bacteria that were frequently identified by their presence and/or abundance in healthy periodontal tissue in the different reviewed studies included those from the genera *Acinetobacter, Actinomyces, Aggregatibacter, Capnocytophaga, Corynebacterium, Gemella, Granulicatella, Haemophilus, Lautropia, Leptotrichia, Neisseria, Porphyromonas, Prevotella, Rothia, Streptococcus,* and *Veillonella.* In particular, the species identified in each of these genera were *Acinetobacter junii, A. antiviralis, A. baumannii, A. calcoaceticus, A. rhizosphaerae, Actinomyces naeslundii, A. viscosus, A. massiliensis, A. meyeri, Aggregatibacter aphrophilus, Capnocytophaga gingivalis, Corynebacterium matruchotti, Gemella morbillorum, Gemella haemolysans, Granulicatella adiacens, Haemophillus parainfluenzae, H. haemolyticus, H. parahaemolyticus, H. sputorum, Lautropia mirabilis, Neisseria lactamica, N. flava, N. subflava, N. oralis, Porphyromonas catoniae, Prevotella histicola, P. nigrescens, Rothia aeria, R. dentocariosa, Streptococcus sanguinis, S. mitis, S. intermedius, S. oralis, S. tigurinus, S. infantis, S. gordonii, Veillonella atypica,* and *V. dispar/V. parvula* (Table 2, Fig. 2).

Bacteria like *A. viscosus, A. naeslundii, G. haemolysans, H. parainfluenzae, P. nigrescens, R. dentocariosa, S. sanguinis, S. mitis, S. oralis, S. gordonii, and S. intermedius* have characteristics like probiotics because they permit modulate the periodontal pathogenic microbiota. However, some strains have been associated with oral or systemic infections, particularly in patients with immune deficiencies. Also, these strains have shown antibiotic resistance (Table 3, Fig. 2).

**Table 3:** Bacteria that were frequently identified by their presence and/or abundance in healthy periodontal tissue and probiotics properties

Bacteria	Reported as bacteria associated with healthy periodontal tissue	Modulates the immune response	Modulates the periodontal pathogenic microbiota	Safe for human consumption
*Acinetobacter junii*	–	–	–	− [33]
*A. antiviralis*	–	–	–	+ [34]
*A. baumannii*	–	–	–	− [35]
*A. calcoaceticus*	–	–	–	− [36]
*A. rhizosphaerae*	–	–	–	+ [37]
*Actinomyces viscosus*	+ [38]	–	+ [38]	− [39, 40]
*A. naeslundii*	–	–	+ [38]	− [41, 42]
*A. massiliensis*	–	–	–	− [43]
*A. odontolyticus/meyeri*,	–	–	+ [38]	− [43]
*Aggregatibacter aphrophilus*	–	–	–	− [44]
*Gemella haemolysans*	–	–	+ [45]	− [46]
*Granulicatella adiacens*	–	–	–	− [47]
*Haemophilus parainfluenzae*	–	–	+ [48]	− [49]
*Lautropia mirabilis*	–	–	–	− [50]
*Leptotrichia*	–	–	–	− [51]
*Neisseria flava*	–	–	–	− [52]
*N. subflava*	–	–	–	− [53]
*N. oralis*	–	–	–	− [54]
*Rothia aeria*	–	–	–	− [55]
*Rothia dentocariosa*	–	–	+ [56]	− [55]
*Streptococcus sanguinis*	–	–	+ [48, 57]	− [58]
*S. mitis*	–	–	+ [48, 59]	− [60]
*S. oralis*	–	–	+ [61]	[62] + [63]
*S. gordonii*	–	–	+ [64]	− [65]
*S. intermedius*	–	–	+ [66]	− [67]
*Porphyromonas catoniae*	–	–	–	–
*Prevotella nigrescens*	–	–	+ [68]	− [69]
*Capnocytophaga gingivalis*	–	–	–	− [70]
*Corynebacterium (C. matruchotti*)	–	–	–	− [71]
*Veillonella atypica*	–	–	–	+ [72],- [73]
*V. dispar/*	–	–	–	− [74]
*V. parvula*	–	–	–	− [75]

### Level of evidence and risk of bias

Based on the JBI tool assessment, most studies met most quality criteria. Only two case–control studies and one cross-sectional study did not meet the identification criteria for confounding factors. Furthermore, the findings of one case–control and two cross-sectional studies were unclear in identifying the confounding factors (Appendix A1 and Appendix A2).

## Discussion

Bacteria must meet certain requirements to be candidates for probiotics, including the ability to colonize the tissues in which they will act [76]; therefore, it is important to identify commensal bacteria that are common or abundant in healthy periodontal tissues [77]. In the present review, we found that both gram-positive and gram-negative bacteria were associated with the subgingival biofilm of healthy periodontium in the reviewed studies. These bacteria mainly comprised facultative anaerobes, including those from the genera *Acinetobacter*, *Actinomyces*, *Aggregatibacter, Capnocytophaga, Corynebacterium, Gemella, Granulicatella, Haemophilus, Lautropia, Leptotrichia, Neisseria, Porphyromonas, Prevotella, Rothia, Streptococcus,* and *Veillonella*.

Furthermore, bacteria of genera, such as *Gemella (G. haemolysans), Granulicatella (G. adiacens), Haemophilus (H. parainfluenzae), Lautropia (L. mirabilis), Neisseria (N. flava, N. subflava,* and *N. oralis), Rothia (R. aeria* and *Rothia dentocariosa), Streptococcus (S. sanguinis, S. mitis, S. oralis, S. gordonii,* and *S. intermedius), Porphyromonas (P. catoniae),* and *Prevotella (P. nigrescens),* were also reported as bacteria specific to patients with healthy periodontium in investigations excluded from this review based on the defined selection criteria [7, 10, 78–83]. In contrast, other genera, such as *Acinetobacter (A. junii, A. antiviralis, A. baumannii, A. calcoaceticus,* and *A. rhizosphaerae*)*,* have been relatively frequently associated with disease conditions [84, 85].

Another frequently reported genus is *Actinomyces* spp., with the species having favorable properties for periodontal health, e.g., *A. viscosus* and *A. naeslundii*, and the species associated with periodontal disease, e.g., *A. massiliensis*, *A. odontolyticus/meyeri*, and, again, *A. naeslundii* [86].

Other bacteria that lead to discrepancies since they are associated with health or disease conditions are *Aggregatibacter* (*A. aphrophilus), Capnocytophaga (C. gingivalis), Corynebacterium (C. matruchotti*), and *Veillonella* (*V*. *atypica*, *V. dispar/V. parvula*) [7, 87]. These discrepancies may be attributed to the fact that the microbiome of healthy periodontal tissue demonstrates greater interpersonal variability than that of patients with periodontitis or gingivitis. This may indicate that the microbiome's composition can be influenced by environmental factors. Other factors that may affect the composition are as follows: diagnosis by selecting healthy controls, which may have caused an imbalance in the microbiota, as such individuals have no clinical symptoms; differences in criteria or clinical measures used to define healthy individuals, as noted in this review; differences in the individual experiences of the examiners; and differences in the sequenced region [8].

Another characteristic of probiotics is the ability to inhibit pathogens by producing antimicrobial agents or competing for nutrients, space, or environmental conditions [88]. Notably, the metatranscriptome analysis of subgingival plaque collected from healthy individuals revealed the expression of genes associated with competition, bacteriocin production (mutation), and quorum sensing [89]. Of the bacteria identified in the present review, *A. viscosus, A. naeslundii, G. haemolysans, H. parainfluenzae, P. nigrescens, R. dentocariosa, S. sanguinis, S. mitis, S. oralis, S. gordonii,* and *S. intermedius* have been reported as those capable of inhibiting the growth of periodontopathogens [50, 68, 90, 91]. For example, *G. haemolysans* produces an inhibitor of *P. gingivalis*; however, it does not inhibit other periodontal pathogens, such as *F. nucleatum* and *T. denticola* [45]. Notably, *H. parainfluenzae* has been reported to have the ability to inhibit the adhesion of periodontopathogenic bacteria, particularly *P. gingivalis* [48]. *Streptococcus* strains secrete substances that cause the lysis of *P. gingivalis* or eliminate its ability to generate a biofilm by affecting the expression of fimA and mfa-1, which are the components of the fimbria of *P. gingivalis* [66]. Regarding *P. nigrescens*, antimicrobial activity has been demonstrated against *Actinomyces* spp., *P. gingivalis*, *P. intermedia*, and *T. forsythensis* by a bacteriocin called nigrescin [68].

Bacteria must also meet other requirements to be candidates for probiotics; these include being harmless, having no pathogenic genes, and showing no characteristics of acquired antibiotic resistance [92, 93]. Some strains of the bacteria that were identified as possible candidates in the present review were found to cause endocarditis and opportunistic infections, as observed with *G. haemolysans* and *H. parainfluenzae* [46, 49]. In addition, *P. nigrescens* belongs to a genus of bacteria associated with pulp infections, periodontitis, and abscesses of dental and periodontal origin, and it is resistant to antibiotics [94]. In a study comparing different species of *Prevotella* (*P. intermedia*, *P. nigrescens*, and *P. melaninogenica*), *P. nigrescens* showed the greatest ability to resist amoxicillin/clavulanic acid, amoxicillin, and clindamycin, and it had more resistant genes to beta-lactams, tetracyclines, and lincosamides [94].

The genus *Streptococcus* includes a wide variety of species previously established as probiotics for human consumption owing to their proven safety [95, 96]. These bacteria are easy to culture under in vitro conditions. For example, the bacterium *S. oralis*, specifically the KJ3 strain, is used in a commercial product with proven safety for human consumption [63]. In addition, *S. mitis* strain YIT 12322 has been studied and found safe for humans [97]. Therefore, *Streptococcus* is a genus with strains such as those identified in the actual review (*S. oralis, S. mitis, S. sanguinis*, and* S. gordonii*) that could be used in future clinical trials. The strain KJ3 of *S. oralis*, along with *S. uberis* strain KJ2 and *S. rattus* strain JH145, constitutes a commercial product named ProBiora®. This product has shown to reduce pathogenic bacteria like *S. mutans*, *Campylobacter rectus*, and *P. gingivalis* in saliva and dental biofilm from young individuals [98]. Another strain of *S. oralis* is the sub-species *dentisani* strain 7746, which can adhere to the tooth to stabilize the pH through arginolytic activities and the production of bacteriocin which inhibits the growth of cariogenic bacteria and periodontopathogens [99]. Through a clinical study, strain 7746 was applied to a dental alginate ferrule every 48 h for one month in patients between 18 and 65 years old. The authors found improved clinical parameters like dental biofilm index, gingival index, and salivary flux [100].

Other bacteria suggested as probiotic candidates are *S. mitis and S. sanguinis,* which are primary colonizers of the oral cavity; these bacteria are characterized by inhibiting the adhesion of *P. gingivalis*, *A. actinomycetemcomitans, P. gingivalis y P. intermedia* to producing H_2_O_2_ [101]. These bacteria were used in replacement therapy for periodontitis in an animal model (dog), where the authors found, after a treatment of 12 weeks, a significant increase in the bone level in the treated periodontal pocket [102].

Finally, another probiotic candidate is *S. gordonii* which promotes the homeostasis of the biofilm through the inhibition of periodontopathogens like *T. denticola*, and *P. gingivalis* [103]. Also, *S. gordonii* causes a beneficial effect on the oral epithelium, stimulating the immune system (activating antigen-presenting cells) [104]. *S. gordonii* can modulate the expression of genes of gingival epithelial cells and decrease the expression of proinflammatory cytokines, indicating an anti-inflammatory potential [105].

However, some strains of the oral streptococci have been isolated from patients with infective endocarditis and other opportunistic infections [106, 107], underscoring the importance of evaluating the safety of bacterial strains as candidate probiotics before conducting clinical trials.

For the prevention or adjuvant treatment of periodontal disease through probiotics, the candidates must be able to maintain or restore the periodontal microecological balance. For this reason, it is necessary to combine several bacterial strains, which, in addition to possessing properties for the control of periodontopathogenic bacteria and regulation of the immune response of the host, comply with the corresponding safety for its use and ease of production at the industrial level.

This study has some limitations. First, not all studies included some factors affecting the microbiota, and therefore, they did not exclude patients who were smokers or pregnant and/or breastfeeding. Second, not all studies used the same number of healthy teeth, similar durations of treatment with antibiotics or other drugs, or similar clinical measures that distinguished between healthy individuals and patients with periodontitis. Finally, although species of bacteria with probiotic properties were identified, not all strains of the same species have the same properties. Nevertheless, this information allows us to focus our studies on the species identified, to isolate and study the most appropriate strains as probiotics.

## Conclusion

There are several important variations in the microbiota associated with healthy periodontal tissue—an area where several of these bacteria have also been reported in disease conditions. This is not only related to the methodological differences between the studies but also to the characteristics of the patients and the nature of their native bacterial strains as well as their virulence factors and the metabolic interactions triggered. Therefore, it is important to reconsider the probiotic strategy because these agents favor the presence of beneficial native bacteria in these tissues.

This study identified bacteria that could be key candidates for maintaining the oral microbiota of periodontal tissues owing to their association with health conditions and their antimicrobial properties. These bacteria include those of the genus *Streptococcus*, such as *S. sanguinis*, *S. oralis,* and *S. mitis*, which have been studied as probiotics for oral health, but these bacterial strains must be selected based on their safety for human consumption.

### Supplementary Information

Additional file1 (DOCX 85 KB)

Additional file2 (DOCX 62 KB)

## Data Availability

All data generated or analyzed during this study are included in this published article and its supplementary information files.
