# Supplementary material for: Bacteria of healthy periodontal tissues as candidates of probiotics: a systematic review
Source: Eur J Med Res. 2024 Jun 14;29:328. doi: 10.1186/s40001-024-01908-2 (PMC11177362; doi:10.1186/s40001-024-01908-2)
Supplement: Supplementary file 1 — Additional file1 (DOCX 85 KB) [file 40001_2024_1908_MOESM1_ESM.docx]

**Appendix A1. Quality criteria for case–control studies.**

|  | | | | | | | | | | |
| --- | --- | --- | --- | --- | --- | --- | --- | --- | --- | --- |
| **ASSESSMENT QUESTION**  **AUTHOR/YEAR** | 1. Were the groups comparable apart from the presence of disease in cases or the absence of disease in controls? | 2. Were the cases and controls matched appropriately? | 3. Were the same criteria used for the identification of both cases and controls? | 4. Was exposure measured in a standard, valid, and reliable manner? | 5. Was exposure measured in the same way for cases and controls? | 6. Were confounding factors identified? | 7. Were strategies to address confounding factors indicated? | 8. Were results evaluated in a standard, valid, and reliable way for both cases and controls? | 9. Was the exposure period of interest long enough to be significant? | 10. Was the statistical analysis adequate? |
| Griffen, et al., 2012 |  |  |  |  |  |  |  |  |  |  |
| Kumar et al., 2012 |  |  |  |  |  |  |  |  |  |  |
| Li et al., 2014 |  |  |  |  |  |  |  |  |  |  |
| Kirst et al., 2015 |  |  |  |  |  |  |  |  |  |  |
| Camelo-Castillo et al., 2015 |  |  |  |  |  |  |  |  |  |  |
| Schulz et al., 2019 |  |  |  |  |  |  |  |  |  |  |
| Ikeda et al., 2020 |  |  |  |  |  |  |  |  |  |  |

| Yes | No | Unclear | Not applicable |
| --- | --- | --- | --- |
