# Supplementary material for: Bacteria of healthy periodontal tissues as candidates of probiotics: a systematic review
Source: Eur J Med Res. 2024 Jun 14;29:328. doi: 10.1186/s40001-024-01908-2 (PMC11177362; doi:10.1186/s40001-024-01908-2)
Supplement: Supplementary file 2 — Additional file2 (DOCX 62 KB) [file 40001_2024_1908_MOESM2_ESM.docx]

**Appendix A2. Quality criteria for cross-sectional studies.**

|  | | | | | | | | |
| --- | --- | --- | --- | --- | --- | --- | --- | --- |
| **ASSESSMENT QUESTION**  **AUTHOR/YEAR** | 1. Were the criteria for inclusion in the sample clearly defined? | 2. Were the study participants and setting described in detail? | 3. Was exposure measured in a valid and reliable way? | 4. Were objective and standardized criteria used to measure the condition? | 5. Were confounding factors identified? | 6. Were strategies to address confounders indicated? | 7. Were outcomes measured in a valid and reliable way? | 8. Was the statistical analysis adequate? |
| Abusleme et al., 2013 |  |  |  |  |  |  |  |  |
| Chen et al., 2018 |  |  |  |  |  |  |  |  |
| López-Martínez et al., 2020 |  |  |  |  |  |  |  |  |
| Lu et al., 2021 |  |  |  |  |  |  |  |  |
| Lenartova et al., 2021 |  |  |  |  |  |  |  |  |

| Yes | No | Unclear | Not applicable |
| --- | --- | --- | --- |
